# Indocyanine Green Loaded Modified Mesoporous Silica Nanoparticles as an Effective Photothermal Nanoplatform

**DOI:** 10.3390/ijms21134789

**Published:** 2020-07-06

**Authors:** Yiyu Wang, Chunqing Niu, Sisi Fan, Yuwei Li, Xiang Li, Yujun Dai, Jian Shi, Xinyu Wang

**Affiliations:** 1 Hubei Province Research Center of Engineering Technology for Utilization of Botanical Functional Ingredients, Hubei Engineering University, Xiaogan 432000, China; niuchunqing@hotmail.com (C.N.); fss18186130620@hotmail.com (S.F.); lyw0213@hotmail.com (Y.L.); lixiang980@hotmail.com (X.L.); dyj5925@hbeu.edu.cn (Y.D.); 2 Hubei Key Laboratory of Quality Control of Characteristic Fruits and Vegetables, Hubei Engineering University, Xiaogan 432000, China; 3 Department of Machine Intelligence and Systems Engineering, Faculty of Systems Science and Technology, Akita Prefectural University, Akita 015-0055, Japan; shij@akita-pu.ac.jp; 4 State Key Laboratory of Advanced Technology for Materials Synthesis and Processing, Wuhan University of Technology, Wuhan 430070, China; 5 Biomedical Materials and Engineering Research Center of Hubei Province, Wuhan University of Technology, Wuhan 430070, China

**Keywords:** mesoporous silica nanoparticles, indocyanine green, NIR-triggered, photothermal effect, tumor cells

## Abstract

Photothermal therapy possesses great advantages for the treatment of drug-resistant tumors. Herein, Near Infrared (NIR)-triggered photothermal nanoparticles were developed through loading indocyanine green (ICG), a kind of NIR dye, into amino group-modified silica nanoparticles (SiO_2_-NH_2_ NPs). SiO_2_-NH_2_ NPs were prepared with immobilization of the amino groups into the framework of silica nanoparticles (SiO_2_ NPs) by employing (3-aminopropyl)-triethoxysilane (APTES). Before and after the modification of the amino group, the particle sizes of SiO_2_ NPs showed similar value, around 100 nm. ICG was further adsorbed into SiO_2_-NH_2_ NPs by electrostatic attraction to enable SiO_2_-NH_2_@ICG NPs as a kind of photothermal agent. The loading rate of ICG to SiO_2_-NH_2_ was greatly increased compared to unmodified SiO_2_, and the stability of ICG was also improved. Moreover, the SiO_2_-NH_2_@ICG NPs exhibited efficient photothermal effects due to ICG transforming laser power into local heat through the connected ICG, when NIR laser irradiation turned on for a couple of minutes. Finally, the in vitro antitumor efficacy of SiO_2_-NH_2_@ICG NPs was investigated by recording cell proliferation rate and further chronicled the apoptotic morphology evidence by a Calcein-AM/PI fluorescent staining assay, indicating the efficient photothermal targeted therapy for the HepG2 tumor cells.

## 1. Introduction

Liver cancer is one of the most common cancers in the world, which is threatening human health and life. In the past few decades, the number of liver cancer patients significantly increased; liver cancer contains hepatocellular carcinoma (HCC) and intrahepatic cholangiocarcinoma (ICC) [1], and HCC patients are often diagnosed at late stages due to its asymptomatic features. Current diagnostic approaches, like ultrasound and α-fetoprotein, are expensive and lack sensitivity in HCC detection [2]. It is necessary to find new systematic therapeutic modalities for diagnosis and therapy of liver cancer. The common cancer therapeutics, such as transarterial chemoembolization, surgical excision of hepatocellular carcinoma and radiofrequency ablation, increase the survival rate of liver cancer patients [3]. However, we still need to study a new therapeutic platform with fewer side effects to break through the bottleneck of traditional therapeutics. Photothermal therapy (PTT) is a minimally invasive technical method for cancers, which can transform the light energy into thermal energy under light irradiation with the help of photoabsorbers [4,5,6]. As is known to all that all light wavelengths can penetrate the skin in different depths, however, Near Infrared (NIR) light can usually penetrate deeper than other wavelengths; therefore, it is more common to use photosensitive nanoparticles in vivo, causing less damage to healthy tissues [7]. Hence, NIR light-triggered photothermal nanoagents have attracted much interest for PTT application. Recently, many photothermal nanoagents were developed for generating efficient thermal energy, such as metal nanoparticles, semiconductor nanoparticles (CuS), boron nitride nanosheets (BN)-polydopamine (PDA) immobilized poly (p-phenylene benzobisoxazole) (PBO) fibers, nanohybrid and carbon-based materials (carbon nanotubes or graphenes) [8,9,10,11,12,13,14].

It is well known to us that various dyes have different and unique light responsiveness and light adsorption, therefore, those dyes can also be applied to various fields [15,16]. Indocyanine green (ICG) is a low toxicity and injectable NIR organic dye; it is also approved by the US Food and Drug Administration (FDA) for clinical use [17,18]. At present, ICG is widely used in clinical diagnosis such as optical angiography [19] and guiding sentinel node biopsy [20]. However, as a small molecular photothermal agent, more extensive applications of ICG are rather limited because of the following drawbacks. ICG has a low fluorescence quantum yield in aqueous solutions because of self-quenching [21] and it cannot be fully used in physiological aqueous conditions [22]. Furthermore, ICG binds to plasma proteins nonspecifically, such as albumin, globulins, and lipoproteins, which can be quickly removed from the circulatory system by the action of the liver, so the initial half-life of pure ICG is only about 3–4 min [23]. To overcome such drawbacks, recently, various types of nanoparticles were adopted for improving the stability of ICG, such as FA ICG-PLGA-lipid NPs, PC-FA/ICG/DOX nanocarriers, MSN-TA-ICG, ICG@HMSNP, and UCNP@mSiO_2_-ICG nanoplatform [24,25,26,27,28,29,30,31]. It was reported that mesoporous silica nanoparticles (SiO_2_ NPs), with ordered mesoporous structure and well-defined surface properties for modification, provided great targeting versatility [32,33,34]. In addition, SiO_2_ NPs have been considered as an excellent ICG carrier due to ease of synthesis, high surface area and pore volume, tunability of their pore size and good stability [35]. Additionally, SiO_2_ has been listed by the US Food and Drug Administration (FDA) as ‘generally recognized as safe’ and is considered to be biocompatible [36,37]. Although PTT and combined PTT and chemotherapy have been developed, the challenge is still ongoing. Therefore, the facile ICG loading to SiO_2_ NPs methods with safe performance need to be further studied for PTT application.

Herein, we designed and fabricated NIR-triggered photothermal NPs by loading the photothermal agent ICG into the amino functional silica nanoparticles (SiO_2_-NH_2_ NPs). SiO_2_-NH_2_ NPs were prepared through an improved base-catalyzed sol–gel method. The characterization of the modified nanoparticles was studied. Then, photothermal ability and ICG stability performance were analyzed. Finally, hemolysis, in vitro cytotoxicity and NIR-triggered phototoxicity, for killing HepG2 tumor cells of the modified nanoparticles, also were fully evaluated.

## 2. Results and Discussion

### 2.1. Preparation and Characterization

**Figure 1:**
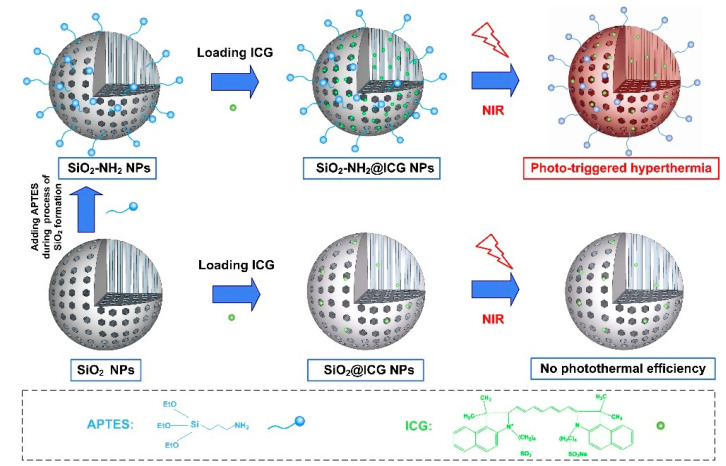
Schematic view of the synthesis procedure of amino group modified SiO_2_ NPs loading with ICG and the NIR-triggered hyperthermia performance.

**Figure 2:**
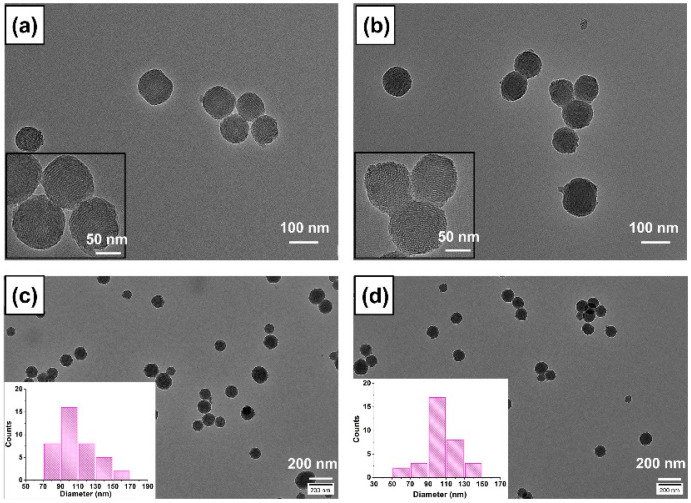
TEM images of (**a**) SiO_2_ NPs (amplified image), (**b**) SiO_2_-NH_2_ NPs (amplified image), (**c**) SiO_2_ NPs (the particle size distribution as an inset), (**d**) SiO_2_-NH_2_ NPs (the particle size distribution as an inset). The histograms were generated by measuring the diameter of about 40 particles from the TEM images.

The synthesis procedure of amino-modified SiO_2_ NPs loading with ICG is schematically elucidated in Figure 1. Firstly, in order to endow amino groups into the framework of SiO_2_ NPs, the modified SiO_2_ NPs were prepared by adding a small amount of aminosilane agent (APTES) during the process of SiO_2_ formation using the sol–gel method. The positively charged SiO_2_-NH_2_ could be later used for static-adsorbing negatively charged ICG molecules. Afterward, ICG was loaded into the SiO_2_-NH_2_ NPs. In this study, ICG was selected as an NIR responsive photothermal agent because the ICG molecule has demonstrated its photothermal effect in terms of its high optical absorbance in the NIR region [27] and it can be used as heat donor for fabrication of NIR-responsive nanoparticle vehicles. We denoted this product as SiO_2_-NH_2_@ICG NPs. The typical outer morphology and inner structure of the SiO_2_ and SiO_2_-NH_2_ were characterized by transmission electron microscope (TEM), as shown in Figure 2. The particle size was calculated by Image J software and particle size distributions are given as insets in Figure 2c,d. These TEM images show both of SiO_2_ and SiO_2_-NH_2_ NPs have spherical particles with uniform size. The average diameter of SiO_2_ measured from TEM images is 108 ± 6 nm, while the diameter of SiO_2_-NH_2_ is around 102 ± 13 nm. The reason for the reduction in particle size of NPs after amino modification may be that the addition of APTES in the final 2 h during the process of SiO_2_-NH_2_ formation had a tiny impact on the deposition of silica. The mesoporous trait of the SiO_2_ is also apparently observed in the SiO_2_-NH_2_, as shown in Figure 2b, and this trait offers the opportunity for the NPs to serve as general drug vehicles. Compared to NPs’ unloaded ICG, the TEM images of NPs after loading ICG do not show any changes in terms of particle size and mesoporous structure inside (Figure S1).

**Figure 3:**
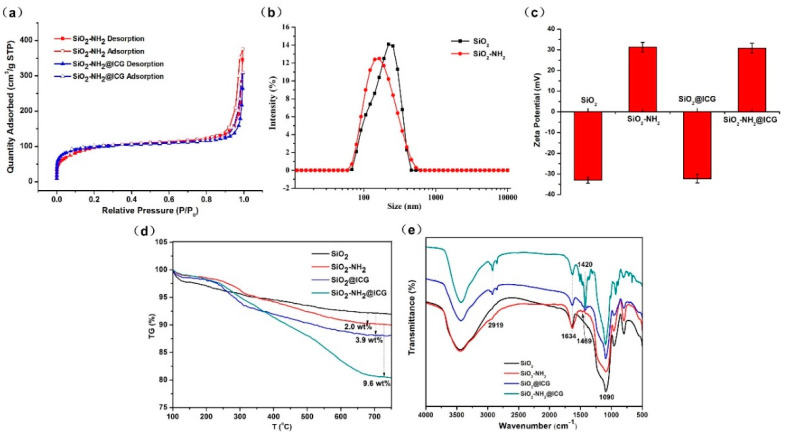
(**a**) N_2_ adsorption/desorption isotherms of the SiO_2_-NH_2_ and SiO_2_-NH_2_@ICG NPs; (**b**) DLS measurement of the size distribution of SiO_2_ and SiO_2_-NH_2_ NPs; (**c**) zeta potentials of SiO_2_, SiO_2_-NH_2_, SiO_2_@ICG and SiO_2_-NH_2_@ICG; (**d**) TG curves of SiO_2_, SiO_2_-NH_2_, SiO_2_@ICG and SiO_2_-NH_2_@ICG NPs; (**e**) Fourier-transform infrared (FTIR) spectra of SiO_2_, SiO_2_-NH_2_, SiO_2_@ICG and SiO_2_-NH_2_@ICG NPs.

The surface area and the inner pore size characteristic of the NPs were analyzed by the N_2_ adsorption/desorption technique, as shown in Figure 3a. SiO_2_-NH_2_ exhibits a reversible type IV isotherm, which is one of the main characteristics of mesoporous materials. The total surface area of SiO_2_-NH_2_ was 426.30 m^2^/g calculated by the (Brunauer–Emmet–Teller) BET model, and the average mesoporous pore diameter of the SiO_2_-NH_2_ was 3.12 nm, which was calculated from the adsorption branch of the isotherm using the Barrett–Joyner–Halenda (BJH) model. After loading ICG, the total surface area slightly decreased to 328.58 m^2^/g and the pore diameter declined to 2.86 nm as well. The result implied this SiO_2_-NH_2_ could be employed as a drug carrier with high drug payload, and reduction in surface area and pore diameter illustrated that ICG can enter the internal channels of SiO_2_-NH_2_ NPs. The DLS results were shown in Figure 3b, which clearly showed both SiO_2_ and SiO_2_-NH_2_ NPs prepared by the sol–gel method exhibited a narrow hydrodynamic particle size distribution. The average hydrodynamic particle sizes of SiO_2_ and SiO_2_-NH_2_ NPs were around 206 ± 15 and 187 ± 10 nm in D.I. water.

The DLS particle size was calculated from the mean data of intensity distribution, which are larger than tested by TEM. The reason for this difference may be ascribed to the hydrodynamic diameter in the hydrated state or aggregation. The results of zeta potential analysis data are shown in Figure 3c. The APTES functionalization containing amino groups changes the zeta potential of the SiO_2_ NPs from a negative to a positive value. The zeta potential of SiO_2_, SiO_2_-NH_2_, SiO_2_-NH_2_, SiO_2_@ICG and SiO_2_-NH_2_@ICG were about −32.95, 31.29, −32.20 and 30.79 mV, respectively. The surface potential of SiO_2_ was successfully changed by adding APTES during the process of SiO_2_ fabrication. ICG loading had little effect on the surface potential of both SiO_2_ and SiO_2_-NH_2_ NPs.

The thermal stability and modification by APTES were examined by TG analysis, as shown in Figure 3d. These NPs showed reliably high stability. Slight weight loss between 100 and 750 °C can be observed, which can mainly be due to the loss of water and hydroxyl groups physically adsorbed in SiO_2_. It could be calculated from the TG curves that the grafted amino group onto SiO_2_ accounted for 2.0 wt%. Furthermore, the weight of ICG loaded into the SiO_2_ and SiO_2_-NH_2_ were measured by TG analysis, and the ICG weight loss from SiO_2_@ICG was calculated to be 3.9 wt%, which is significantly less than 9.6 wt% from SiO_2_-NH_2_@ICG, indicating that ICG loading capacity of SiO_2_-NH_2_ was significantly higher than that of SiO_2_. The FTIR spectra were presented in Figure 3e. The FTIR spectra of pure ICG were also recorded in the SI Figure S2. As shown in Figure 3e, for SiO_2_ (black curve), the peaks around 1090 and 800 cm^−1^ are assigned to characteristic absorption peaks of silica. The absorption peak at 3400 cm^−1^ can be attributed to the stretching of -OH and the peaks around 1634 cm^−1^ belong to the bending of H–O–H [38]. The red curve of Figure 3e represents SiO_2_-NH_2_; comparing to curve of SiO_2_, a new weak absorption peak at 1469 cm^−1^ appeared, which can be ascribed to characteristic absorption peaks of amino group in the modifier [39]. As can be seen from SiO_2_@ICG (blue curve) and SiO_2_-NH_2_@ICG (green curve), new absorption peaks appeared at 2919 and 1420 cm^−1^, assigned to characteristic peaks of ICG, indicating that the both SiO_2_ and SiO_2_-NH_2_ NPs were successfully loaded with ICG. Moreover, these two absorption peaks were much stronger in the green curve, further confirming SiO_2_-NH_2_ possessed a higher loading rate for ICG.

### 2.2. Loading of ICG Molecules and Photothermal Effect

**Figure 4:**
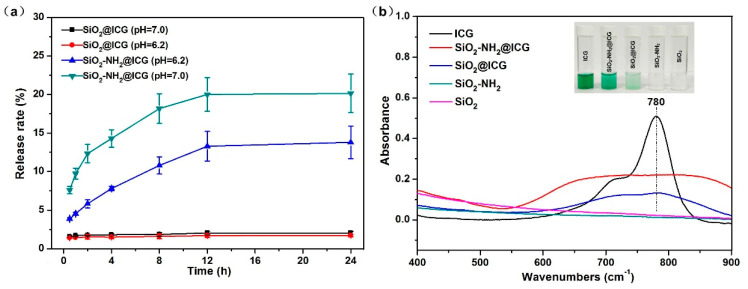
(**a**) Release behavior of ICG from the SiO_2_@ICG NPs and SiO_2_-NH_2_@ICG NPs in PBS (pH = 6.2 or pH = 7.4); (**b**) UV–vis spectra of ICG, SiO_2_-NH_2_@ICG, SiO_2_@ICG, SiO_2_-NH_2_ and SiO_2_ NPs in aqueous solution (ICG concentration is 5 μg/mL, NPs concentration is 500 μg/mL).

ICG were loaded into the SiO_2_ and SiO_2_-NH_2_ NPs via small molecule free diffusion and intermolecular forces between ICG and functional groups of the NPs surface. The molecular weight of ICG is 548.63, which is small enough to load into the framework of SiO_2_-NH_2_ NPs [28]. ICG loading experiment results showed that the ICG loading efficiency (LE)of SiO_2_ NPs was relatively low, only 21.60%, while the ICG LE of SiO_2_-NH_2_ NPs was significantly improved to 99.87% (Table S1). According to the previous reports [28], modified mesoporous silica with the TA group into the framework can adsorb more ICG by strong electrostatic attraction between sulfonic groups of ICG and amino groups of modified silica. SiO_2_-NH_2_ NPs showed obvious positive zeta potential due to amino modification, so SiO_2_-NH_2_ NPs can be employed as an ICG carrier with high loading amount. The ICG release rates from the SiO_2_@ICG and SiO_2_-NH_2_@ICG in vitro were shown in the Figure 4a. Almost no premature ICG release from SiO_2_@ICG was observed after 48h in PBS solution. In addition, the ICG release from SiO_2_-NH_2_@ICG was also relatively low, which finally reached 20.14% (pH = 7.0) after 24h and remained a stable value with extension of time. Under weak acid condition, ICG release was significantly reduced to 13.80% (pH = 6.2), because pH value affects the electrostatic interaction between ICG and the NPs [28]. The lower ICG release rate in vitro indicated that ICG loaded into nanoparticles could be stable for use as a photothermal agent.

UV–vis spectra of the obtained drug loaded NPs and the pure NPs were monitored through the UV–vis analyzer, as shown in Figure 4b. The absorption spectrum of ICG showed a clear characteristic peak around 780 nm. In addition, the spectra of ICG loaded NPs in an aqueous solution showed relatively broader and lower between 600 and 950 nm compared with that of free ICG, which meant that the ICG molecules were actually inside the NPs [40]. The absorption spectrum of SiO_2_-NH_2_@ICG was obviously stronger than that of SiO_2_@ICG, which confirmed higher ICG loading capacity of SiO_2_-NH_2_ NPs once again. The broader absorption band of NPs might be attributed to the polaronic-π* transitions because of the electrostatic interaction between ICG molecules and the NPs [28,41,42].

**Figure 5:**
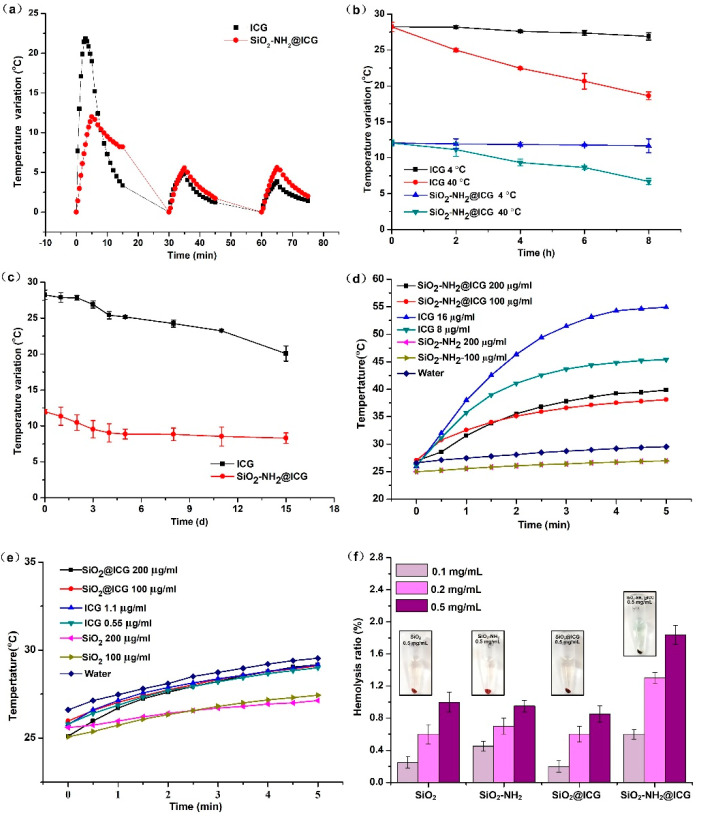
(**a**) Irradiation stability of SiO_2_-NH_2_@ICG. (**b**) Thermal stability of SiO_2_-NH_2_@ICG. (**c**) Long-term stability of SiO_2_-NH_2_@ICG. (**d**) Temperature elevation of SiO_2_-NH_2_@ICG NPs with different concentrations with exposure to NIR laser irradiation (808 nm, 1.5 W/cm^2^) for 5 min. DI water was used as a control. (**e**) Temperature elevation of SiO_2_@ICG NPs with different concentrations with exposure to NIR laser irradiation (808 nm, 1.5 W/cm^2^) for 5 min. DI water was used as a control. (**f**) Hemolysis ratio of nanoparticles with different concentrations.

As an NIR dye, the stability of ICG greatly affects its photothermal performance. Free ICG are labile in aqueous solution and are easily prone to degradation and destruction. However, the stability of ICG can be significantly improved by nanocarrier encapsulation. In this study, irradiation stability, thermal stability and long-term stability were measured, as shown in Figure 5a–c. When under irradiation of an NIR laser the first time, the temperature rise of ICG solution was evidently higher than that of the SiO_2_-NH_2_@ICG NPs group. The reason for this difference of temperature changes is that the peak absorbance of ICG in nanoparticle suspension was about half that of the free ICG solution, and this attenuation of absorbance can be responsible for the lower photothermal effect when the samples were irradiated for the first time. However, as the irradiation times increased, the temperature variation of ICG solution decreased obviously, while the NPs group remained the relatively stable photothermal efficiency compared to free ICG solution. After the third irradiation, the photothermal ability of the two groups were nearly equal. The irradiation stability results indicated that the photothermal ability of the SiO_2_-NH_2_@ICG NPs group was more stable after multiple irradiations, because free ICG suffer degradation and destruction when exposed to constant lights [43]. Figure 5b showed that the photothermal effect for both of samples were almost unchanged within 8 h at low temperature, while the photothermal effect decreased gradually with the extension of storage time at 40 °C, and the photothermal effect of ICG solution decreased more obviously. Therefore, low temperature was beneficial to maintain the photothermal efficiency for both of them, and free ICG became more unstable under high temperature conditions, but nanoparticles entrapped ICG was to establish its effectiveness in preserving ICG from thermal damage. Furthermore, after 15 days of storage at 4 °C (Figure 5c), photothermal efficiency of SiO_2_-NH_2_@ICG NPs had remained stable since the third day, while the photothermal efficiency of the ICG solution dropped continuously.

The photothermal effect of ICG, SiO_2_-NH_2_@ICG and SiO_2_@ICG was investigated using D. I. water as a blank control. It can be observed from Figure 5d that the temperature of ICG solution increased fastest from 27 to 54 °C. However, temperature variation under NIR irradiation was approximately halved in the case of SiO_2_-NH_2_@ICG compared to free ICG. This may be due to the significantly lowered absorbance of SiO_2_-NH_2_@ICG in the NIR region. Besides, with the increasing ICG or SiO_2_-NH_2_@ICG concentration, the temperature rose much faster upon NIR irradiation. It can be seen from the Figure 5e that the photothermal efficiency of SiO_2_@ICG was much lower under NIR irradiation. The different temperature increase in Figure 5d,e was due to the much less amount of ICG entrapped inside SiO_2_ when the concentration of NPs was same. The hemolysis assays were used to evaluate the biocompatibility of these NPs. The results in Figure 5f showed that the maximum hemolysis ratio was at 1.84 ± 0.12% assigned to 0.5 mg/mL of SiO_2_-NH_2_@ICG NPs, indicating that the red blood cells are not compromised even at relatively high concentration (0.5 mg/mL). The hemolysis ratios of all groups were far less than 5%, which implied that they possessed good blood compatibility.

### 2.3. In Vitro Cytotoxicity and Phototoxicity Study

**Figure 6:**
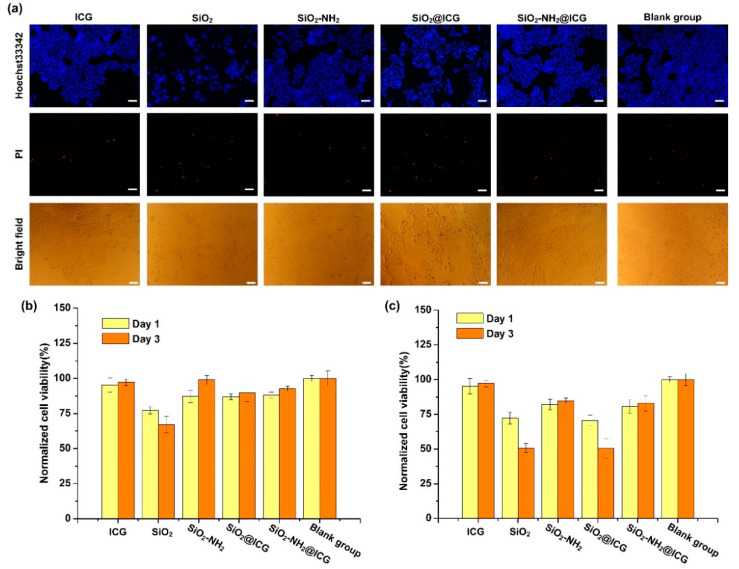
(**a**) The HepG2 cells treated with free ICG or different samples media after the 3-day culture were stained with Hoechst 33342 (blue) and PI (red) fluorescent dye (ICG concentration was fixed at 8 μg/mL), scale bars, 100 μm. (**b**) Viability of HepG2 cells on the first and third days after incubation with ICG (4 μg/mL) or different samples media (50 μg/mL) but without laser irradiation. (**c**) Viability of HepG2 cells on the first and third days after incubation with ICG (8 μg/mL) or different samples media (100 μg/mL) but without laser irradiation.

To intuitively evaluate the cytotoxicity of NPs aforementioned, Hoechst 33342 and the PI fluorescent staining method was used to confirm apoptosis and cell death. Figure 6a showed photomicrographs of HepG2 cells stained with Hoechst 33342 (blue) and PI (red) fluorescent dye after exposure to free ICG and different NPs groups for three days. Compared to the blank group, a similar number of normal cells were observed in the ICG, SiO_2_-NH_2_, and SiO_2_-NH_2_@ICG groups. Meanwhile, there were relatively higher dead cells observed in the SiO_2_ and SiO_2_@ICG groups, indicating that both SiO_2_ and SiO_2_@ICG NPs exhibited higher cytotoxicity than the other NPs.

In order to further analyze the effect of NPs on cell apoptosis, Annexin V-FITC/PI staining assay was performed to confirm the apoptosis of HepG2 cells. The apoptosis of HepG2 cells exposure to SiO_2_, SiO_2_-NH_2_, SiO_2_@ICG, and SiO_2_-NH_2_@ICG without NIR treatment was investigated by Flow Cytometry, while cells co-incubated with normal medium and free ICG were used as controls (the concentration of ICG was fixed at 8 μg/mL). As shown in Figure S3 and Table S2 (Supporting Information), the cells treated different NPs did not display significant apoptosis, whereas the cells treated with SiO_2_ exhibited relatively higher apoptosis rate, at 4.59%. The apoptosis rates of all groups were less than 5%, and negligible necrosis also was observed, which indicated free ICG and NPs obtained in this experiment cannot induce cell apoptosis. These results could be ascribed to the fine biocompatibility of unirradiated ICG and the superior cellular compatibility of SiO_2_-NH_2_.

The viability results of HepG2 cells exposure to the free ICG or NPs media with different concentrations were recorded in the Figure 6b,c. It can be seen from Figure 6b that the viability of HepG2 cells incubation with NPs media at the 50 μg/mL for one and three days were insignificant difference compared to the blank group, except in the SiO_2_ group. A slight reduction in cell viability after incubation with SiO_2_ for three days can be observed. Moreover, as the concentration of NPs rose to 100 μg/mL, viability of HepG2 cells incubation with NPs media declined at different levels. The SiO_2_-NH_2_ and SiO_2_-NH_2_@ICG groups exhibited higher HepG2 cells proliferation compared with the SiO_2_ and SiO_2_@ICG groups. Significantly reduced cell viability of 50% after incubation with SiO_2_ and SiO_2_@ICG for three days was observed, indicating SiO_2_ and SiO_2_@ICG showed the higher cytotoxicity to HepG2 cells. These results indicated that the SiO_2_-NH_2_ and SiO_2_-NH_2_@ICG NPs with better cellular compatibility could promote HepG2 cell proliferation.

**Figure 7:**
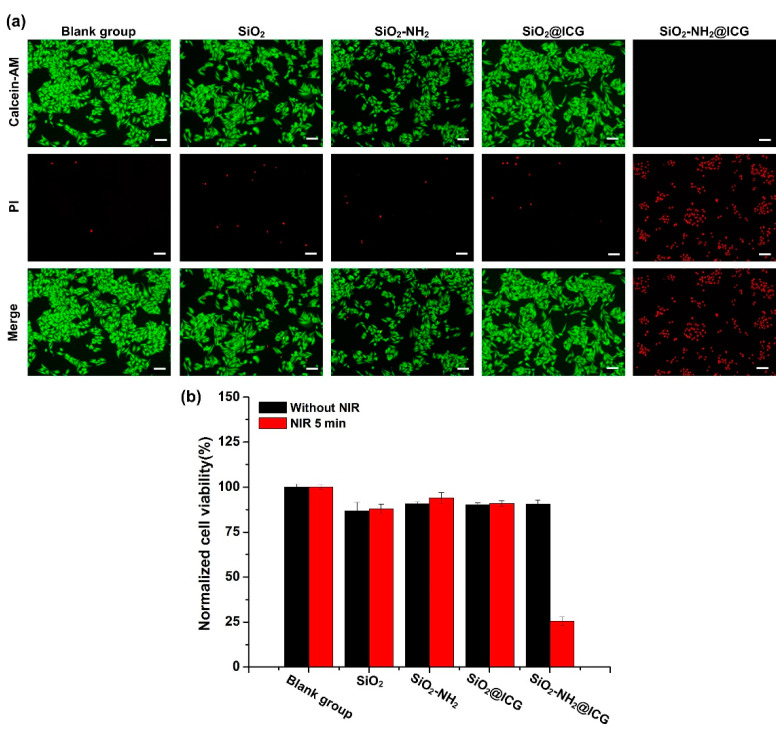
(**a**) The HepG2 cells treated with different samples media after NIR irradiation 5 min; after further 12 h culture, the cells were stained with Calcein-AM/PI fluorescent dye, scale bars, 100 μm. (**b**) Viability of HepG2 cells treated with SiO_2_, SiO_2_-NH_2_, SiO_2_@ICG and SiO_2_-NH_2_@ICG NPs, with or without NIR irradiation (808 nm, 1.5 W/cm^2^, 5 min).

To evaluate the NIR responsive photothermal effect of SiO_2_-NH_2_@ICG for killing HepG2 cells, Calcein-AM and PI fluorescent staining and MTT assay were applied to distinguish the live/dead cells and evaluate cells viability with or without NIR irradiation. Figure 7a showed there were no obvious dead cells that could be observed in the SiO_2_, SiO_2_-NH_2_, SiO_2_@ICG and blank group under NIR irradiation, suggesting that 808 nm NIR laser illumination with the output of 1.5 W cm^−2^ for 5 min was safe for the HepG2 cells, and SiO_2_@ICG had inefficient photothermal effect due to the low ICG loading rate, which cannot kill HepG2 cells. However, the cells incubated with SiO_2_-NH_2_@ICG were induced to death obviously under NIR irradiation, indicating it possessed superior photothermal conversion ability that can kill HepG2 cells effectively.

As shown in Figure 7b, all NPs groups showed high cell viability after 16 h incubation period without irradiation even at a high concentration of 200 μg/mL (equivalent 16 μg/mL ICG). The viability of HepG2 cells incubated with SiO_2_, SiO_2_-NH_2_, and SiO_2_@ICG did not change whether with a laser or not, whereas the HepG2 cells treated with SiO_2_-NH_2_@ICG saw a sharp decrease in viability to 25%. This result confirmed that SiO_2_-NH_2_@ICG had low cytotoxicity in HepG2 cells, but can effectively induce cell death via photothermal effects of plenty of encapsulated ICG.

## 3. Materials and Methods

### 3.1. Materials

Tetraethoxysilane (TEOS), (3-aminopropyl)-triethoxysilane (APTES), tetyl trimethylammonium bromide (CTAB), and indocyanine green (ICG) were purchased from Shanghai Aladdin Bio-Chem Technology Co. Ltd. (China). DMEM (Gibco, Grand Island, NY, USA), Trypsin (HyClone, UT, USA), Penicillin/Streptomycin (HyClone) and Fetal Bovine Serum (FBS, Gibco) were purchased from Shanghai Pufei Bio-Technology Co. Ltd. (China). Hoechst 33342 and PI Assay Kit, Annexin V-FITC and PI Assay Kit, 3-(4,5-dimethyl -2-thiazolyl)-2,5-diphenyl -2-H-tetrazolium bromide (MTT), Calcein-AM/PI Double Stain Kit and Dimethyl sulfoxide (DMSO) were purchased from Beijing Solarbio Science & Technology Co., Ltd. (China). A HepG2 cell line was provided from the American Type Culture Collection (ATCC, USA). 

### 3.2. Synthesis of the SiO_2_-NH_2_ NPs

The amino-functionalized silica nanospheres were prepared by the sol–gel method using CTAB as a template according to the published literature [9]. Briefly, 0.279 g of CTAB was dissolved in 200 mL of DI water at 50 °C with stirring on the magnetic stirrer, and 6 mL of aqueous ammonia was added dropwise. Then, 1.394 mL TEOS was slowly added into the mixtures and stirred at 600 r/min for 4 h. Next, 0.3 mL of APTES were further introduced into the mixtures, followed with another 2 h stirring until white precipitates formed. The precipitates were washed twice with DI water and anhydrous ethanol, respectively. After cleaning, in order to remove the template (CTAB), the precipitation was refluxed at 80 °C for 36 h in acidic methanol (100 mL of methanol added to 2 mL of HCl). After reflux, the precipitates were centrifuged at 10,000 r/min for 10 min, and then, washed twice with DI water and anhydrous ethanol, respectively. Finally, the products were dried in the vacuum drying chamber overnight and recorded as SiO_2_-NH_2_ NPs. The unmodified silica nanoparticles were prepared in the same way without adding APTES and recorded as SiO_2_ NPs. The final products were stored in a vacuum for further study.

### 3.3. Preparation of the ICG-Loaded Nanoparticles

To load ICG into the SiO_2_-NH_2_ and SiO_2_ NPs, 3 mL of SiO_2_-NH_2_ NPs (5 mg/mL in water) and 3 mL ICG solution (400 µg/mL in water) were mixed; this solution was oscillated at 25 °C at 110 r/min for 2 h. The precipitates were collected by using centrifugation at 8000 r/min for 5min and then, washed three times to remove the unloaded ICG. The ICG loaded NPs were then freeze-dried and stored in the dark. The quantity of loaded ICG was measured using an EnSpire Microplate Reader (PerkinElmer, Waltham, MA, USA) at 780 nm. The ICG LE was calculated according to Equation.

(1) $$LE\;(\%)=\frac{\mathrm{weight}\;\mathrm{of}\;\mathrm{loaded}\;\mathrm{drug}}{\mathrm{initial}\;\mathrm{weight}\;\mathrm{of}\;\mathrm{drug}\;}\times 100$$

### 3.4. Characterization

The morphologies of the samples were characterized using a TEM images obtained on a JEM-1200EX microscope (JEOL, Japan), operating at 120 kV. Image-Pro plus soft was used to measure the average particle size. UV–vis spectra were acquired on an EnSpire Microplate Reader. FTIR spectra were recorded using a Nicolet 6700 IR spectrometer (Thermo Fisher, Waltham, MA, USA). Dynamic light scattering (DLS) studies were carried out using Zetasizer Nano ZS ZEN3600 (Malvern, UK) at 25 °C to analyze particle size distribution and zeta potential. The specific surface area and mesoporous pore diameter of the product were measured by nitrogen adsorption method using the ASAP 2020M specific surface area and pore size distribution analyzer (Micromeritics, Norcross, GA, USA). Thermogravimetric analysis was performed with a Netzsch STA 409 thermogravimetric analyzer (Netzsch, Germany) in the air, and the heating rate was 10 °C min^−1^.

In order to study the release behavior of ICG from the SiO_2_-NH_2_@ICG and SiO_2_@ICG NPs, 600 μg samples were suspended in 3 mL PBS solution (0.01 mM, pH = 6.2 and pH = 7.0) at 37 °C, respectively. At specific time points, the suspension was centrifuged to separate the NPs precipitate and the released ICG into supernatant, respectively. The absorbance of the supernatants was measured using a Microplate Reader. Three parallel samples were prepared for each group.

### 3.5. Measurement of the Photothermal Performance

The photothermal performance was examined by observing the temperature elevation of the different concentrations of different kinds of NPs suspension with NIR laser irradiation. Briefly, 1.0 mL of SiO_2_-NH_2_@ICG suspension with different concentrations (100 and 200 μg/mL) were put into a glass cuvette (optical path is 1 cm) and then, irradiated by an 808 nm high power diode laser with an output of 1.5 W/cm^2^ for 5 min, and dimensions of the beam at the aperture were 5 × 8 mm^2^. The temperature elevation of the solutions was recorded by a TES-1310 Thermometer (Taiwan, China) with a thermocouple microprobe submerged in the solution, while the direct irradiation of the laser on the probe was avoided. Temperature elevations of free ICG, SiO_2_@ICG, SiO_2_-NH_2_, and SiO_2_ NPs suspension with the same concentration were measured simultaneously using the same methods. A total of 1 mL of DI water was used as the blank control. The equivalent concentrations of ICG solution for 100 and 200 μg/mL SiO_2_-NH_2_@ICG suspension were 8 and 16 μg/mL, and these concentrations for 100 and 200 μg/mL SiO_2_@ICG suspension were 1.1 and 0.55 μg/mL.

### 3.6. Measurement of ICG Stability Performance

#### 3.6.1. Irradiation Stability

In total, 200 μg/mL of SiO_2_-NH_2_@ICG suspension were prepared; 1 mL suspension was put into a glass cuvette, and then, irradiated by an 808 nm NIR laser with an output of 1.5 W/cm^2^ for 3 times in a cycle. The irradiation lasted for 5 min each time and the interval was 30 min. The temperature elevation of the solution was also recorded by a TES-1310 Thermometer during the experiment. The ICG solution with equivalent concentration was used as the control (16 μg/mL). The experiment was carried out in three parallel groups.

#### 3.6.2. Thermal Stability

An amount of 200 μg/Ml of SiO_2_-NH_2_@ICG suspension was prepared. ICG solution with equivalent concentration was used as a control (16 μg/Ml). All the samples were stored in an oven at 40 °C and a fridge at 4 °C without light irradiation, respectively. After 2, 4, 6 and 8 h, 1 Ml of uniform suspension was put into a glass cuvette, and then, irradiated only one time by an 808 nm NIR laser with an output of 1.5 W/cm^2^ for 5 min; the temperature elevation of the solution was recorded by a TES-1310 Thermometer. Samples at each time point were freshly unirradiated suspensions and solutions. The experiment was carried out in three parallel groups. 

#### 3.6.3. Long-Term Stability

In total, 200 μg/mL of SiO_2_-NH_2_@ICG suspension and ICG solution with equivalent concentration were prepared (16 μg/mL). All the samples were kept in a 4 °C refrigerator avoiding light. At some specific time points, 1 mL was sampled in the glass cuvette, and then, irradiated by an 808 nm NIR laser with an output of 1.5 W/cm^2^ for 5 min, and the temperature elevation was recorded. The experiment was carried out in three parallel groups.

### 3.7. Hemolysis Test

A total of 1 mL fresh rabbit blood was taken and washed with 0.9% normal saline. Red blood cells were diluted with 10 mL normal saline. The NPs suspensions with different concentrations (0.1, 0.2 and 0.5 mg/mL) were prepared, and all the NPs suspension and 0.9% normal saline were kept at 37 °C for 30 min. Then, 0.2 mL of diluted red blood cells were added in 0.8 mL NPs suspension; after mixing thoroughly, the mixture was incubated at 37 °C for 60 min. Following centrifugation, the supernatant was taken and the absorbance of the supernatant at the wavelength of 570 nm was measured by a Microplate Reader. The experiment was carried out in three parallel groups. DI water was used as positive control and 0.9% normal saline as negative control. The hemolysis rate was calculated according to Equation (2):

(2) $$Hemolysisrate\;(\%)=\frac{{\mathrm{OD}}_{\mathrm{sample}}-{\mathrm{OD}}_{\mathrm{negative}\;\mathrm{group}}}{{\mathrm{OD}}_{\mathrm{positive}\;\mathrm{group}}-{\mathrm{OD}}_{\mathrm{negative}\;\mathrm{group}}\;}\times 100$$

### 3.8. Cytotoxicity Assay

To investigate the cytotoxicity of SiO_2_, SiO_2_-NH_2_, SiO_2_@ICG and SiO_2_-NH_2_@ICG, HepG2 cells were fed regularly with DMEM media supplemented with 10% FBS and 1% streptomycin–penicillin in a humidified incubator at 37 °C and 5% CO_2_. Cells were plated in 96-well tissue culture plates at a density of 1 × 10^4^ cells/well the day before experiments. Then, SiO_2_, SiO_2_-NH_2_, SiO_2_@ICG and SiO_2_-NH_2_@ICG suspensions were added at two kinds of concentrations (50 and 100 μg/mL) to the cells and were incubated for 24 or 96 h, respectively. Free ICG solutions with equivalent concentrations (4 and 8 μg/mL) were obtained by diluting ICG in cell culture medium. At each determined time point, cell viability was evaluated by MTT according to the manufacturer’s specification. The normalized viability was denoted as the percentage of the cell viability relative to the negative control. The cells were stained with fluorescence dye (Hoechst 33342 and PI) at day 3, and then, were observed by a fluorescence microscope (Olympus, IX71, Japan). 

### 3.9. Cell Apoptosis Assay

HepG2 cells were plated in 6-well plates at a density of 2 × 10^5^ cells per well and incubated for 24 h. Then, the cells were exposed to free ICG, SiO_2_, SiO_2_-NH_2_, SiO_2_@ICG and SiO_2_-NH_2_@ICG (the concentration of ICG was fixed at 8 μg/mL, and the concentration of NPs was fixed at 100 μg/mL). After incubation together for 48 h, the cells were washed with PBS several times and digested by trypsin, collected by centrifugation and washed twice. Then, the cells were resuspended in 0.5 mL of annexin-binding buffer and stained with Annexin V-FITC and PI, before testing by Flow Cytometry (Cytoflex, Beckman, Brea, CA, USA).

### 3.10. In Vitro Phototoxicity Testing

To determine the photothermal effect, cells were plated in 96-well polystyrene plates at a density of 1 × 10^4^ cells/well the day before experiments, then blank NPs and ICG-loaded NPs (200 μg/mL) were added to the cells and incubated for 4 h, following irradiation by an 808 nm NIR laser with an output of 1.5 W/cm^2^ for 5 min. The cells that were incubated with the same samples for 4 h but were not under irradiation were used as the control. After another 12 h incubation, the cells were washed and stained with Calcein-AM/PI according to the manufacturer’s protocol and recorded by a fluorescence microscope.

Blank NPs and ICG-loaded NPs (200 μg/mL) were incubated with cells for 4 h; the cells in the NIR-treated groups were exposed to the same 808 nm laser for 5 min. After another 12 h incubation, the cell viabilities were evaluated using MTT assay. The normalized viability was denoted as the percentage of the cell viability relative to the negative control.

## 4. Conclusions

In summary, an NIR-triggered photothermal nanoparticle has been designed and prepared successfully via the modified sol–gel method, and then, through loading enough contents of ICG into SiO_2_-NH_2_ NPs. Due to the change of surface potential, the loading capacity of SiO_2_-NH_2_ NPs with positive zeta potential for ICG was greatly increased, and endowed SiO_2_-NH_2_@ICG NPs capable of effective photothermal conversion. Furthermore, after encapsulation of ICG, the absorption range of SiO_2_-NH_2_@ICG NPs in the NIR region became broader, indicating this type of nanoparticle is suitable to be used as NIR-triggered photothermal nanocarriers. As expected, under NIR irradiation, the SiO_2_-NH_2_@ICG NPs showed effective photothermal performance. Thanks to the protection of SiO_2_-NH_2_ NPs, the stability of ICG improved effectively, and the slow and continuous release of ICG from SiO_2_-NH_2_ NPs endowed this nanoparticle with slower decay properties and longer photothermal effect. Finally, the cytotoxicity and phototoxicity study results demonstrated the SiO_2_-NH_2_@ICG NPs exhibited the highest phototoxicity to HepG2 cells compared to other treatments under NIR irradiation. This study demonstrated that this NIR-triggered photothermal SiO_2_-based nanoparticle could provide a potential platform for enhanced cancer cell killing.

## Supplementary Materials

Supplementary Materials can be found at https://www.mdpi.com/1422-0067/21/13/4789/s1. The following are available online: Figure S1: TEM images of (a) SiO_2_ NPs loaded ICG (average diameter is 107 nm), (b) SiO_2_-NH_2_ NPs loaded ICG (average diameter is 102 nm). Figure S2: FTIR spectra of ICG; Figure S3: The HepG2 cell apoptosis after treatment with (a) normal media, (b) SiO_2_, (c) SiO_2_-NH_2_, (d) SiO_2_@ICG, (e) SiO_2_-NH_2_@ICG NPs, (f) free ICG. ICG concentration was fixed at 8 μg/mL, NPs concentration was fixed at 100 μg/mL.; Table S1: ICG loading efficiency (LE) of SiO_2_ and SiO_2_-NH_2_; Table S2: HepG2 cell apoptosis results.
