# Supplementary material for: Indocyanine Green Loaded Modified Mesoporous Silica Nanoparticles as an Effective Photothermal Nanoplatform
Source: Int J Mol Sci. 2020 Jul 6;21(13):4789. doi: 10.3390/ijms21134789 (PMC7369735; doi:10.3390/ijms21134789)
Supplement: Supplementary file 1 [file ijms-21-04789-s001.pdf]

Supplementary Materials:

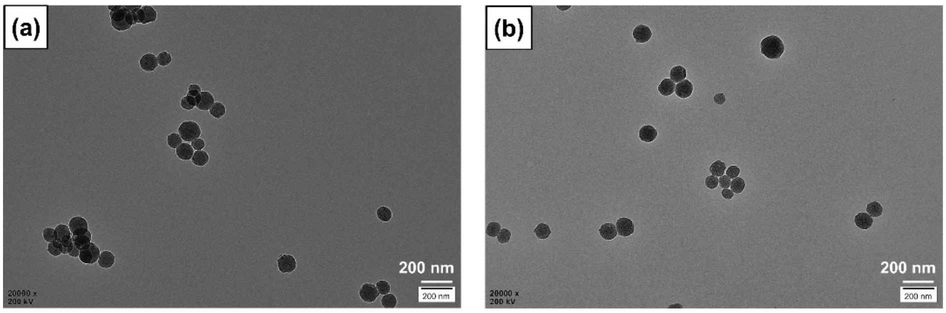

Figure S1. TEM images of (**a**) SiO_2 NPs loaded ICG (average diameter is 107 nm), (**b**) SiO_2-NH_2 NPs loaded ICG (average diameter is 102 nm).

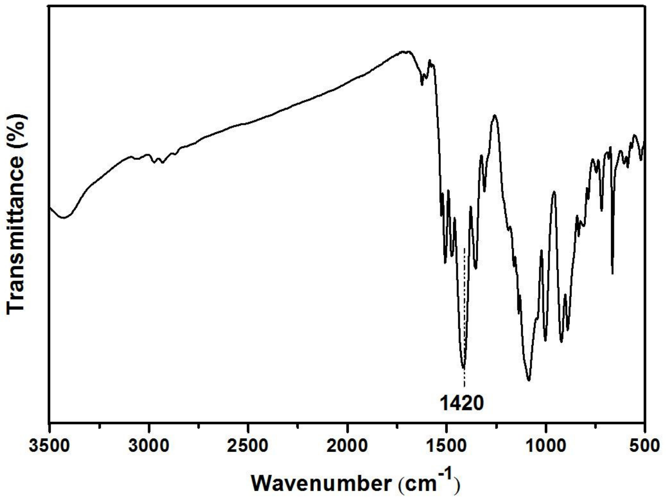

Figure S2. FTIR spectra of ICG.

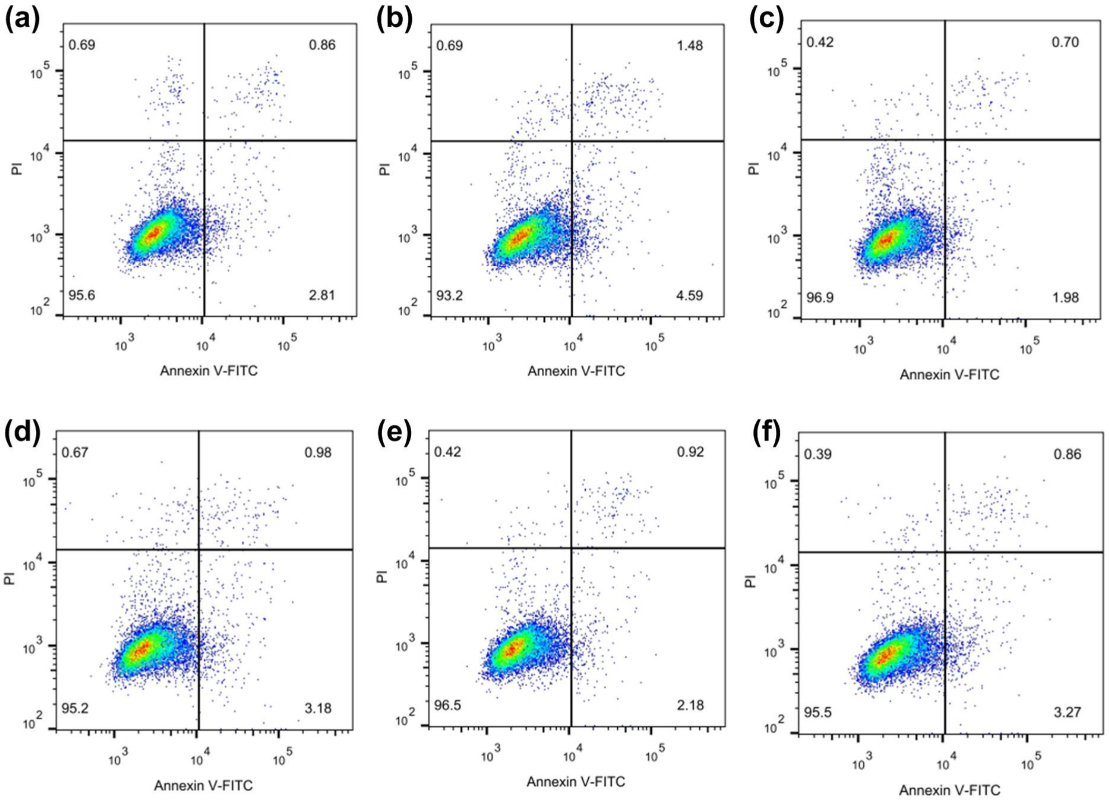

Figure S3. The HepG2 cell apoptosis after treatment with (**a**) normal media, (**b**) SiO_2, (**c**) SiO_2-NH_2, (d) SiO_2@ICG, (e) SiO_2-NH_2@ICG NPs, (f) free ICG. ICG concentration was fixed at 8 μg/mL, NPs concentration was fixed at 100 μg/mL.

Table S1. ICG loading efficiency (LE) of SiO2 and SiO2-NH2.

Sample	SiO_2	SiO_2-NH_2
LE (%)	21.60 ± 1.5	99.87± 0.7

Table S2. HepG2 cell apoptosis results.

Sample	Normal Media	SiO_2	SiO_2-NH_2	SiO_2@ICG	SiO_2-NH_2@ICG	Free ICG
Apoptosis rate (%)	2.81 ± 0.12	4.59 ± 0.22	1.98 ± 0.09	3.18 ± 0.30	2.18 ± 0.19	3.27 ± 0.19
Necrosis rate (%)	0.86 ± 0.07	1.48 ± 0.10	0.70 ± 0.03	0.98 ± 0.11	0.92 ± 0.08	0.86 ± 0.05
Live cell rate (%)	95.6 ± 3.40	93.2 ± 3.10	96.9 ± 2.52	95.2 ± 4.17	96.5 ± 3.23	95.5 ± 2.74
